# Evaluation of Fatty Acid Compositions, Antioxidant, and Pharmacological Activities of Pumpkin (*Cucurbita moschata*) Seed Oil from Aqueous Enzymatic Extraction

**DOI:** 10.3390/plants10081582

**Published:** 2021-07-31

**Authors:** Adchara Prommaban, Ratthida Kuanchoom, Natthidaporn Seepuan, Wantida Chaiyana

**Affiliations:** 1 Department of Pharmaceutical Sciences, Faculty of Pharmacy, Chiang Mai University, Chiang Mai 50200, Thailand; waewaa@gmail.com (A.P.); ratthida_k@elearning.cmu.ac.th (R.K.); nutty_eve@outlook.co.th (N.S.); 2 Research Center of Pharmaceutical Nanotechnology, Chiang Mai University, Chiang Mai 50200, Thailand; 3 Innovation Center for Holistic Health, Nutraceuticals and Cosmeceuticals, Faculty of Pharmacy, Chiang Mai University, Chiang Mai 50200, Thailand

**Keywords:** *Cucurbita moschata*, Japanese pumpkin, pumpkin seed oil, aqueous enzymatic extraction, fatty acids, antioxidant, anti-aging

## Abstract

Pumpkin seed oil is a by-product, abundant in nutrients and bioactive components that promote several health benefits. This study aimed to compare chemical compositions, antioxidant, and pharmacological activities of pumpkin seed oils extracted from *Cucurbita moschata* Duch. Ex Poir. (PSO1) and *Cucurbita moschata* (Japanese pumpkin) (PSO2) by aqueous enzymatic extraction. An enzyme mixture consisting of pectinase, cellulase, and protease (1:1:1) was used in the enzymatic extraction process. Fatty acid composition of the oils was determined using fatty acid methyl ester/gas chromatographic-mass spectrometry. Antioxidant activity assays were measured by using stable free radical diphenylpicrylhydrazyl, radical cation 2,2′-azinobis-(3-ethylbenzothiazoline-6-sulfonate, ferric reducing/antioxidant power, and ferric thiocyanate assay. Inhibition of enzymes involving skin aging and whitening process was investigated. Linoleic acid was a major component of all pumpkin seed oils. Additionally, there was also a significant amount of oleic acid, palmitic acid, and stearic acid detected. PSO2 possessed the highest antioxidant activities compared to PSO1 and commercial pumpkin seed oils (COM1 and COM2). Both PSO1 and PSO2 exhibited higher inhibitory effects on hyaluronidase, collagenase, and tyrosinase than the commercials. Therefore, aqueous enzymatic extraction could yield pumpkin seed oils with higher antioxidant, anti-aging, and whitening activities. This is beneficial for further pharmacological studies and can be used as a functional food for skin benefits.

## 1. Introduction

Pumpkin is in genus Cucurbita and the family Cucurbitaceae, a common and famous plant which was cultivated from northern Mexico and has spread to Europe, Western America, and Asia [1]. Pumpkin is considered as a functional food which contains abundant nutrients such as protein, carbohydrate, lipid, fiber, etc., along with the phytochemical compounds such as tocopherols, carotenoids, and β-sitosterol [2]. The phytonutrients from various parts of pumpkin have been found to have pharmacological activities [2]. Pumpkin peel extract showed beneficial burn wound effect in animal models [3]. Pumpkin pulp demonstrated antioxidant, anti-inflammation, anti-angiogenesis activity [2], as well as anti-fatigue activity in mice [4]. In addition, pumpkin seed is a good natural source of essential fatty acid and phytosterols which lower risk of cardiovascular mortality [5,6]. Pumpkin seed oil is a by-product from the processing of pumpkin seeds, which are rich in various fatty acids and bioactive compounds such as β-carotenes, α-tocopherol, vitamin B, lutein, phytosterols, and other minerals [7,8]. The oil exerts antioxidant, anti-inflammation, antibacterial, and wound healing effects [1,9]. Moreover, it has several health benefits to against diseases, for instance hypertension, diabetes, and cancer [8,10].

Different methods for extracting seed oil have been developed, including mechanical methods such as cold-pressing [11] and high-pressure extraction [12]. Solvent extraction of seed oil using organic solvents such as hexane, ethyl acetate, acetone, and methanol has also been documented [13,14]. Enzyme-assisted extraction is an alternative method for the plant oil industry because it is an environmentally friendly and safe technology. The major mechanism of aqueous enzymatic extraction is hydrolyzing and breaking the cell walls of plant material, leading to more permeability and releasing of bioactive components, including the oil phase [15]. A variety of enzyme mixtures applied in this extraction are composed of pectinases, cellulases, hemicellulases, arabanase, β-glucanase, and xylanase [16,17]. The optimized conditions of an enzyme mixture, for example temperature, pH, reaction time, particle size, and enzyme concentration, also enhanced enzyme activity [16]. Nowadays, this method has been widely used to extract oil from many fruits and plant seeds [18,19]. Some previous studies demonstrated the optimized condition of pumpkin seed oil to obtain high yield and effectively pharmacological activities [20,21]. 

Currently, people consume pumpkin seed oil in desserts or salad dressings, and it is also a popular ingredient in cosmetics. Skin aging is a complex biological process which is characterized by wrinkling, loss of elasticity, loss of moisture, and rough texture caused by oxidative stress, DNA damage, and advanced glycation end product accumulation [22]. Hyaluronic acid, collagen, and elastin, which are important components of skin, can be degraded by hyaluronidase, collagenase, and elastase enzymes, respectively, resulting in skin aging. Several plant oils, such as olive oil, sunflower seed oil, grape seed oil, and jojoba oil have been used for skin cosmetic properties to retain moisture, improve skin barrier function, and prevent skin aging [23]. Additionally, skin pigmentation is a variable and noticeable phenotype in humans which involves melanogenesis and is regulated by the tyrosinase enzyme [24]. At present, various plant extracts have been tested for cosmetics and pharmaceuticals to decrease melanin production, acting as whitening agents [25,26]. 

Recently, *C. moschata* Duch. Ex Poir is one pumpkin cultivar which is commonly cultivated and popular in Thailand, but there are few studies involving its functional values and pharmacological properties. Furthermore, aqueous enzymatic extraction is an interesting method to extract oil from pumpkin seeds in order to obtain high yield and efficiency. Therefore, this study focused on the determination of main constituents, antioxidant, and pharmacological activities of pumpkin seed oil from *C. moschata* Duch. Ex Poir. (Thai cultivar) and *C. moschata* (Japanese pumpkin)using the aqueous enzymatic extraction method.

## 2. Materials and Methods

### 2.1. Plant Materials

The fresh fruits of C. moschata Duch. Ex Poir and C. moschata (Japanese pumpkin) were purchased from a local market in Chiang Mai, Thailand during December 2020. The seeds were collected and all fibrous strands were removed. After the seed coat elimination, the hulless pumpkin seeds were washed and dried at an ambient temperature. The seeds were kept in a sealed plastic bag until further experimentation. Additionally, two commercial pumpkin seed oil samples (COM1 and COM2) were purchased from a supermarket in Chiang Mai, Thailand. All experiments were performed within the date of suitability for consumption of the commercial oils.

### 2.2. Chemicals and Reagents

Collagenase from *Clostridium histolyticum* (ChC—EC.3.4.23.3), elastase from porcine pancreas (PE—E.C. 3.4.21.36), N-succinyl-Ala-Ala-p-nitroanilide, ferric sulfate (FeSO_4_), hyaluronic acid sodium salt from *Streptococcus equi*, hyaluronidase from bovine tests, pectinase from *Aspergillus niger* (EC 3.2.1.15, ≥5 units/mg protein, optimum pH = 4.0, optimum temperature = 61 °C), cellulase from *Aspergillus niger* (EC 3.2.1.4, ≥0.3 units/mg solid, optimum pH = 6.5, optimum temperature = 50–55 °C), protease from *Aspergillus saitoi* (EC 3.4.21.112, ≥0.6 units/mg solid, optimum pH = 5.1, optimum temperature = 57.2 °C), 2,2′-azinobis 3-ethylbenzothiazoline-6-sulphonate (ABTS), 2,2-diphenyl-1-picrylhydrazyl-hydrate (DPPH), 2,4,6 tripyridyl-s-triazine (TPTZ), 6-hydroxy-2,5,7,8-tetramethylchroman-2-carboxylic acid (Trolox), bovine serum albumin, kojic acid, L-tyrosine, L-DOPA, linoleic acid, sodium phosphate monobasic (NaH_2_PO_4_∙2H_2_O), sodium phosphate dibasic, and tyrosinase from mushroom were purchased from Sigma-Aldrrich (St. Louis, MO, USA). Additionally, 37% hydrochloric acid, 95% ethanol, distilled (DI) water, dimethyl sulfoxide (DMSO) and methanol were purchased from RCI Labscan Co., Ltd. (Bangkok, Thailand). Ammonium thiocyanate (NH_4_SCN) and ferrous chloride (FeCl_2_) were purchased from Loba Chemie Pvt. Ltd. (Mumbai, India). Tris (hydroxymethyl) aminomethane was purchased from Merck (Darmstadt, Germany). 

### 2.3. Aqueous Enzymatic Extraction

Pumpkin seed oils from both *C. moschata* Duch. Ex Poir and *C. moschata* (Japanese pumpkin) were extracted by aqueous enzymatic extraction method using the enzymatic hydrolysis parameters from a previous study by Kanopka et al. (2016), including enzyme concentration, enzyme to substrate ratio, reaction temperature, pH, and reaction time [21]. Three different types of enzymes, including pectinase, cellulase, and protease, were combined in a 1:1:1 weight ratio to generate a concentrate cocktail of enzymes. Thereafter, the resulting concentrate cocktail was diluted in DI water to generate a 2% *w*/*w* aqueous enzymatic mixture. The hull-less pumpkin seeds were then added to the resulting aqueous enzymatic mixture in a weight ratio of 1:1. The mixture was then finely ground using a Moulinex DB81 blender at room temperature until homogeneity. The pH of the resulting slurry was then adjusted to 4.7 using 0.1 M HCl and incubated at 54 °C for 15 h. After the slurry was cooled down to room temperature, it was centrifuged at 11,500× *g* for 10 min. The upper layer of supernatants, which was the oil phase, was collected. The cream produced during the extraction process was not demulsified but removed by siphoning using a micropipette. The residue of pumpkin seed was removed by filtration through Whatman No.1 filter paper under vacuum [21]. Pumpkin seed oil from *C. moschata* Duch. Ex Poir (PSO1) and *C. moschata* (Japanese pumpkin) (PSO2) was kept in well-closed, light-resistant containers at room temperature until further experimentation.

### 2.4. Determination of Chemical Composition of Pumpkin Seed Oil Using Fatty Acid Methyl Ester/Gas Chromatographic-Mass Spectrometric (FAME/GC/MS) Method

Pumpkin seed oil was determined for fatty acid composition by using the fatty acid methyl ester/gas chromatographic-mass spectrometric (FAME/GC/MS) method. FAME is a type of fatty acid ester that is derived by acid-catalyzed transesterification of fats with methanol. In this study, pumpkin seed oils (PSO1, PSO2, COM1 and COM2) were saponified by adding 0.5 M NaOH solution in methanol at 100 °C for 15 min. After cooling down, the oil samples were derivatized with boron trifluoride (BF_3_) in methanol solution at 100 °C for 1 min to form FAME products. After cooling and the addition of saturated NaCl and hexane, the FAME were partitioned into hexane phase and were collected into vials for injection. The FAME extracts were analyzed by using GC/MS technique under the following conditions: capillary column dimension (TR-FAME, size 60 m × 0.25 mm, 0.25-µm particle size), running temperature program of 50 °C; hold 2 min with ramp 1 rate of 10 °C/min up to 180 °C, hold 15 min; ramp 2 rate of 4 °C/min, final temperature 230 °C, hold 22.50 min. Helium was used as carrier gas at a flow rate of 1.0 mL/min. The oil samples were injected in a splitless mode (split ratio 1:20 and split flow 20 mL/min) at 235 °C by an autosampler. An MS-detector fitted with an electrospray ionization (ESI) source operated at 70 eV with the temperatures of the ion source and the transfer line set at 200 °C and 220 °C, respectively, recorded mass spectra in the *m*/*z* range of 38–600. Analyzed compounds were assigned by comparing their mass spectra with published data (National Institute of Standards and Technology, 2008). Percent composition was calculated by integrating peaks in total ion chromatograms (TIC). All measurements were performed by the Research and Service Laboratory, The Halal Science Center, Chulalongkorn University, Bangkok, Thailand under the Halal GMP/HACCP and Halal-QHS/ISO 22000.

### 2.5. Determination of Antioxidant Activities

#### 2.5.1. 2,2-Diphenyl-1-Picrylhydrazyl (DPPH) Radical Scavenging Assay

DPPH radical (DPPH^•^) scavenging activity was determined by using the method established by Chaiyana et al. (2017) [27]. In brief, 20 µL of oil samples (PSO1, PSO2, COM1, COM2) and linoleic acid dissolved in DMSO were mixed with 180 µL of 167 µM DPPH ethanolic solution in a 96-well plate and then incubated for 30 min at room temperature in the dark. Ascorbic acid (1 mg/mL) was used as a positive control. The optical density (OD) was measured at 520 nm by using a microplate reader (Microplate readers EZ Read 2000, Biochrome, England). The experiments were done in triplicate and performed in three independents. The percentage of DPPH inhibition was calculated using Equation (1):

DPPH inhibition (%) = {[(OD_A_ − OD_B_) − (OD_C_ − OD_D_)]/(OD_A_ − OD_B_)} × 100, (1)

where OD_A_ is the OD of mixture containing DI water and DPPH^•^ solution; B is the OD_B_ of mixture containing DI water and DMSO; C is the OD_C_ of mixture containing oil samples and DPPH^•^ solution; and OD_D_ is the OD of mixture containing oil samples and DMSO.

#### 2.5.2. 2,2′-Azino-Bis-3-Ethylbenzthiazoline-6-Sulphonic Acid (ABTS) Assay

ABTS cationic radical (ABTS^•+^) scavenging activity was measured by colorimetric method according by Chaiyana et al. (2017) and Paradee et al. (2019) [27,28] with slight modification. ABTS^•+^ was generated by adding 2 mL of 7 mM ABTS with 3 mL of 2.45 mM potassium persulfate and incubated for 16–24 h at room temperature in the dark. The resulting ABTS^•+^ was subsequently diluted with ethanol to obtain an OD of 0.7 ± 0.1 at 750 nm. Briefly, 20 µL of oil samples (PSO1, PSO2, COM1, COM2) and linoleic acid dissolved in DMSO were mixed with 180 µL of ABTS^•+^ ethanolic solution in a 96-well plate and incubated for 5 min at room temperature. The OD was measured at 750 nm by using a microplate reader (Microplate readers EZ Read 2000, Biochrome, England). Ascorbic acid (1 mg/mL) was used as a positive control. The standard curve was produced by plotting the absorbance at 750 nm versus different concentrations of Trolox ranging from 2.5–30 µg/mL. ABTS^•+^ scavenging activity was calculated from Trolox standard curve (R^2^ = 0.9922) and expressed as Trolox equivalent antioxidant capacity (TEAC). The experiments were done in triplicate and performed in three independents.

#### 2.5.3. Ferric Reducing Antioxidant Power (FRAP) Assay

Ferric reducing antioxidant power (FRAP) of each of the samples was determined using FRAP assay as described a previous study by Chaiyana et al. (2017) which had been slightly modified from Saeio et al. (2011) [27,29]. FRAP reagent was freshly generated by mixing of 300 mM acetate buffer (pH 3.6), 10 mM 2,4,6 tripyridyl-s-triazine (TPTZ) in 40 mM HCl, and 20 mM FeCl_3_ in the proportion of 10:1:1. In this study, 20 µL of oil samples (PSO1, PSO2, COM1, COM2) and linoleic acid were mixed with 180 µL of FRAP reagent in 96-well plate and incubated for 5 min at room temperature in dark. The OD was measured at 595 nm by using a microplate reader (Microplate readers EZ Read 2000, Biochrome, England). Ascorbic acid (1 mg/mL) was used as a positive control. The standard curve was produced by plotting the absorbance at 595 nm versus different concentrations of ferric sulfate (FeSO_4_) ranging from 0.003–0.5 mM. FRAP value was calculated from FeSO_4_ standard curve (R^2^ = 0.9965) and expressed as equivalent capacity (EC_1_). The experiments were done in triplicate and performed in three independents.

#### 2.5.4. Ferric Thiocyanate (FTC) Assay

The inhibition of the lipid peroxidation was investigated by the ferric thiocyanate (FTC) method. This method was performed according to the steps described by Osawa et al. (1981) [30] with slight modification. The mixture was composed of 50 µL of oil samples (PSO1, PSO2, COM1, COM2) and linoleic acid, 50 µL of 50% linoleic acid in DMSO, 50 µL of 10% ammonium thiocyanate (NH_4_SCN) aqueous solution, and 50 µL of 2 mM ferrous chloride (FeCl_2_). Following, the mixture was incubated at 37 ± 2 °C for 1 h and OD measured at 500 nm by using a microplate reader (Microplate readers EZ Read 2000, Biochrome, England). α-Tocopherol (0.025–0.1 mg/mL) was used as a positive control. The experiments were done in triplicate and performed in three independents. The lipid peroxidation inhibition activity was calculated using Equation (2):

Lipid peroxidation inhibition (%) = {[(OD_A_ − OD_B_) − (OD_C_ − OD_D_)]/(OD_A_−OD_B_)} × 100, (2)

where OD_A_ is the OD of the mixture containing linoleic acid, NH_4_SCN, and FeCl_2_; OD_B_ is the OD of DMSO; OD_C_ is the OD of the mixture containing oil samples, linoleic acid, NH_4_SCN, and FeCl_2_; and OD_D_ is the OD of the mixture containing oil samples and DMSO.

### 2.6. Determination of Anti-Aging Activities

#### 2.6.1. Anti-Hyaluronidase Activity

Anti-hyaluronidase activity measurement was performed as previously described by Chaiyana et al. (2020) [31]. First, 20 µL of oil samples (PSO1, PSO2, COM1, COM2) were mixed with 100 µL of 15 U/mL hyaluronidase solution and incubated at 37 °C for 10 min. After the incubation, 100 µL of 0.03% *w*/*v* hyaluronic acid which was dissolved in 20 mM phosphate buffer (pH 5.35) was added, then incubated at 37 °C for 45 min. After that, 1 mL of 0.1% bovine serum albumin was added and incubated at room temperature for 10 min. The OD was determined at 600 nm using a multimode detector (Beckman Coulter DTX880, Fullerton, CA, USA). Oleanolic acid (0.25% *w*/*v*) was used as a positive control. The experiments were done in triplicate and performed in three independents. Percentage of hyaluronidase inhibition was calculated using Equation (3):

Hyaluronidase inhibition (%) = {[(OD_A_ − OD_B_) − (OD_C_ − OD_D_)]/(OD_A_ − OD_B_)} × 100, (3)

where OD_A_ is the OD of the mixture containing DI water, hyaluronidase solution, and hyaluronic acid; OD_B_ is the OD of the mixture containing DI water and phosphate buffer (pH 5.35); OD_C_ is the OD of the mixture containing oil samples, hyaluronidase solution, and hyaluronic acid; and OD_D_ is the OD of the mixture containing oil samples and phosphate buffer (pH 5.35).

#### 2.6.2. Anti-Collagenase Activity

Anti-collagenase activity measurement was performed according to the process established by Chaiyana et al. (2019) and Thring et al. (2009) with slight modification [32,33]. Briefly, 20 µL of oil samples (PSO1, PSO2, COM1, COM2) were mixed with 20 µL of 0.1 U/mL collagenase solution from Clostridium histolyticum and incubated at 37 °C for 15 min. After the incubation, 80 µL of 50 mM tricine buffer (400 mM NaCl and 10 mM CaCl_2_, pH 7.5) and 40 µL of N-[3-(2-furyl) acryloyl]-Leu-Gly-Pro-Ala (FALGPA) substrate solution were added. The OD was immediately detected at 340 nm and then continuously measured for 20 min by using a microplate reader (Microplate readers EZ Read 2000, Biochrome, England). Oleanolic acid (1% *w*/*v*) was used as a positive control. The experiments were done in triplicate and performed in three independents. Percentage of collagenase inhibition was calculated using Equation (4):

Collagenase inhibition (%) = [(OD_A_ − OD_B_)/OD_A_] × 100, (4)

where OD_A_ is the OD of the mixture containing oil samples, collagenase solution, tricine buffer and FALGPA substrate; and OD_B_ is the OD of the mixture containing DMSO, collagenase solution, tricine buffer, and FALGPA substrate.

#### 2.6.3. Anti-Elastase Activity

Anti-elastase activity measurement was performed according to the process established by Chaiyana et al. (2019) and Thring et al. (2009) with slight modification [32,33]. Briefly, 10 µL of oil samples (PSO1, PSO2, COM1, COM2) were mixed with 40 µL of 0.03 U/mL elastase solution from porcine pancreas, then 50 µL of 200 mM Tris-HCL buffer (pH 8.0) and 100 µL of 0.8 mM N-Succinyl-Ala-Ala-Ala-p-nitroanilide (AAAPVN) substrate solution were added. The OD was immediately detected at 410 nm and then continuously measured for 20 min by using a microplate reader (Microplate readers EZ Read 2000, Biochrome, England). Oleanolic acid (1% *w*/*v*) was used as a positive control. The experiments were done in triplicate and performed in three independents. Percentage of elastase inhibition was calculated using Equation (5):

Elastase inhibition (%) = [(OD_A_ − OD_B_)/OD_A_] × 100, (5)

where OD_A_ is the OD of the mixture containing oil samples, elastase solution, Tris-HCl buffer, and AAAPVN substrate; and OD_B_ is the OD of the mixture containing DMSO, elastase solution, Tris-HCl buffer, and AAAPVN substrate.

### 2.7. Determination of Anti-Tyrosinase Activities

Whitening effect of a natural extract was established by anti-tyrosinase activity measurement which was performed according to Saeio et al. (2011) and Laosirisathian et al. (2020) [29,34]. L-tyrosine and L-DOPA were the substrates of tyrosinase enzyme in this study. In brief, 10 µL of oil samples (PSO1, PSO2, COM1, COM2) were mixed with 30 µL of tyrosinase in a 96-well plate, then incubated at room temperature for 10 min. After incubation, 100 µL of substrate, which are 2.5 mM of L-tyrosine or L-DOPA, was added and incubated for 30 min. The OD was determined at 450 nm using a microplate reader (Microplate readers EZ Read 2000, Biochrome, England). Kojic acid (20 µg/mL) was used as a positive control. The experiments were done in triplicate and performed in three independents. Percentage of tyrosinase inhibition was calculated using Equation (6):

Tyrosinase inhibition (%) = {[(OD_A_ − OD_B_) − (OD_C_ − OD_D_)]/(OD_A_ − OD_B_)} × 100, (6)

where OD_A_ is the OD of the mixture containing DMSO, tyrosinase enzyme, and L-tyrosine; OD_B_ is the OD of the mixture containing tyrosinase enzyme and PBS with a pH of 6.8; OD_C_ is the OD of the mixture containing oil samples, tyrosinase enzyme, and L-tyrosine; and OD_D_ is the OD of the mixture containing oil samples, tyrosinase enzyme, and PBS with a pH of 6.8.

### 2.8. Statistical Analysis

All data were demonstrated as a mean ± standard deviation (SD). Statistical significance was analyzed using one-way analysis of variance (ANOVA) followed by Tukey’s post-hoc test using GraphPad Prism (version 8.0, GraphPad Software), and *p* < 0.05 was considered statistically significant.

## 3. Results and Discussion

### 3.1. Pumpkin Seed Oil Character

In this study, we used aqueous enzymatic extraction instead of organic solvent extraction to extract pumpkin seed oil from C. moschata Duch. Ex Poir (PSO1) and C. moschata (PSO2) because it is a simple, effective, and productive process [15,21]. PSO1 was a dark-brown liquid with low viscosity, whereas, PSO2 was a greenish-brown liquid with low viscosity. Both pumpkin seed oils had their own characteristic odor. The yields of PSO1 and PSO2 were 17.7% *v*/*w* and 15.6% *v*/*w*, respectively. Apart from the oil, pumpkin seed has been reported to contain a variety of nutritional compounds. The proximate compositions of C. moschata were mainly carbohydrate (39.51%), followed by fat (28.49%), protein (19.23%), water (7.67%) and ash (5.18%), respectively [35]. However, as compared to the previous studies using solvent and mechanical pressing, the present study revealed lower yields of C. moschata oil. Although solvent extraction has been described as the most effective oil extraction method, extracting up to 98% oils from seeds, there are some issues concerning solvent residues and the purity of the produced oil [36]. Additionally, the solvent employed in the extraction process had an effect on the yield of the generated oil. Extraction with hexane, petroleum ether, petroleum benzene, cyclohexane, isopropyl ether, ethyl acetate, tetrahydrofuran, propan-2-ol, and acetone yielded 43.4–64.4% [37,38]. Therefore, cold-press was more preferable than the solvent extraction. However, a cold-press may cause some complexities in the initial stage of using a screw press [39]. Therefore, aqueous enzymatic extraction could be an alternative extraction method since it yielded higher pumpkin seed oil content (36.0%) when compared to cold-pressed oil (33.5%) [21]. Although the process of aqueous enzymatic extraction was more complicated than cold-press, it comprises a relatively cheaper investment cost, a continuing decrease in the cost of commercial enzyme preparations, a potential to simultaneously isolate unique and valuable phytochemical components, as well as addressing the widespread desire for the implementation of green technologies [21]. Preliminary studies described that several enzymes such as cellulases, pectinases, hemicellulase, and protease have been often used to destroy plant cell wall structure to enhance bioactive extraction from plants [16,17]. Therefore, we designed this study to extract seed oil by using an enzyme mixture containing pectinase, cellulase, and protease (1:1:1) as previously described by Kanopka et al. (2016), who optimized the enzymatic hydrolysis conditions to obtain the highest yield of pumpkin seed oil [21]. Furthermore, this method shows safer and more environmentally sustainable alternatives to solvent extraction, here termed “green extraction”.

### 3.2. Chemical Composition of Pumpkin Seed Oil

For fatty acid composition analysis, pumpkin seed oil using aqueous enzymatic extraction (PSO1 and PSO2) was compared and analyzed together with commercial pumpkin seed oil (COM1 and COM2) produced by cold-press extraction. The fatty acid compositions of PSO1, PSO2, COM1 and COM2 are shown in Table 1. All pumpkin seed oil samples consisted of polyunsaturated (PUFA), monounsaturated (MUFA), and saturated fatty acids. Cis-linoleic acid (C18:2) was the main composition in all oil samples. COM2 contained the highest content of fatty acid (51.74%), followed by COM1 (48.00%), PSO1 (39.09%), and PSO2 (37.63%), respectively. Similarly, cis-oleic acid (C18:1) was present in high percentage, especially in PSO2 (37.45%), followed by COM1 (33.39%), PSO1 (31.22%), and COM2 (28.64%). Saturated fatty acids such as palmitic acid (C16:0) and stearic acid (C18:0) were found in only small amounts in each of the oil samples. Trace amounts of other PUFA, MUFA, and saturated fatty acids were also observed and identified.

In literatures, linoleic acid is also known as omega-6, an essential fatty acid which cannot be synthesized by the human body, only received by dietary consumption. Linoleic acid is an important nutrient for health functions in humans, involving a precursor to ceramides which is a major component of cellular membranes, vitamin D and a variety of hormones [40,41]. Animal studies have shown that a linoleic acid deficiency can cause scaly and itchy skin [23]. Additionally, linoleic acid from plant oil has supported skin wound healing, along with preventing skin inflammation and acne [23]. Moreover, oleic acid, or omega-9, has been beneficial in preventing cancer, autoimmune and inflammatory diseases [42]. Indeed, oleic acid significantly reduced nitric oxide production at the wound site, resulting in faster wound closure [43]. These data support that pumpkin seed oil, which enriches to linoleic acid (omega-6) and oleic acid (omega-9), exhibits potential health benefits.

From our findings, pumpkin seed oil (PSO1, PSO2, COM1 and COM2) consisted of many fatty acids including linoleic acid (C18:2), oleic acid (C18:2), palmitic acid (C16:0) and stearic acid (C18:0) which are common components found in pumpkin seed oil [44]. Linoleic acid (omega-6) and oleic acids (omega-9) were dominant in all oil samples. The highest amount of linoleic acid was COM2, followed by COM1, PSO1 and PSO2, respectively. Also, the highest amount of oleic acid was PSO2, followed by COM1, PSO1 and COM2. These results explain that aqueous enzymatic extraction of pumpkin seed oil had a slightly lower linoleic acid, but not oleic acid than cold-press extraction. The reason for the lower content of linoleic acid in pumpkin seeds from aqueous enzymatic extraction than in commercial oil samples was due to a difference in the extraction process. Previous studies have found inconsistencies in the amount of unsaturated fatty acids, especially oleic acid and linoleic acid, in pumpkin seed oils from distinct extractions [39]. The content of oleic acid ranged from 28.19% in cold-pressed pumpkin seed oil to 30.56% in pumpkin seed oil extracted by pentane, whereas the content of linoleic acid ranged from 43.86% in pumpkin seed oil extracted by pentane to 46.67% in cold-pressed pumpkin seed oil [39]. Apart from different extraction methods, the solvents used in the extraction process and the temperature during seed maturation also affected the linoleic acid content of plant seeds. A previous study highlighted that petroleum ether yielded linseed oil with low linoleic content (26.2%), while *n*-hexane yielded contradicting results with a linoleic acid content of 46.5% [45]. Additionally, linoleic acid is inversely proportional to temperature during sunflower seed maturation [46].

In addition, a relative study discovered that the chemical composition in terms of fatty acid composition revealed that linoleic and oleic acids were present in 47.45% and 35%, respectively, in pumpkin seed oil (*C. maxima*) extracted using diethyl ether [47]. According to Akin et al. (2018), the content of linoleic acid ranged from 53.19% to 53.27%, followed by oleic acid, which was also present in high amounts ranging from 27.52% to 27.59% in cold-pressed *C. pepo* L. seed oil extraction [48]. Therefore, aqueous enzymatic extraction in this study also obtained lower yields of fatty acids than solvent extraction and cold-press extraction mentioned in previous studies. In cultivar comparison, we found that PSO1 (*C. moschata* Duch. Ex Poir) showed higher amount of linoleic acid than PSO2 (*C. moschata,* or, Japanese pumpkin). Diversely, PSO2 presented higher amount of oleic acid than PSO1. Importantly, variations in fatty acid profiles and amounts of pumpkin seed oil were dependent on the origin of particular cultivars, climatic conditions, and post-harvest management [49]. Apart from differences in fatty acid profiles of pumpkin seed oils obtained from different extraction methods, the oils from aqueous enzymatic technology were reported to be rich in phytosterol and tocopherol [50]. Therefore, PSO1 and PSO2, which were produced from the green extraction method, are suggested as alternative pumpkin seed oils for further applications.

### 3.3. Antioxidant Activities of Pumpkin Seed Oil

**Figure 1:**
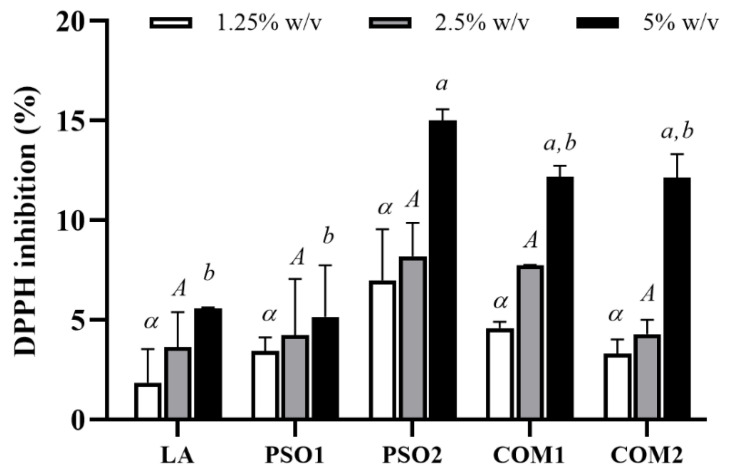
DPPH^●^ inhibitory effect of pumpkin seed oil samples (PSO1, PSO2, COM1, COM2) and linoleic acid (LA). Data obtained from triplicate and three independent experiments are expressed as mean ± SD. The symbol, *α*, presents no significant difference among samples at the concentration of 1.25% *w*/*v*. The capital letter, *A*, presents no significant difference among samples at the concentration of 2.5% *w*/*v*. The letters, *a* and *b*, present significant differences among samples at the concentration of 5% *w*/*v*, *p* < 0.05.

All pumpkin seed oil samples reduced DPPH^●^ radicals in a dose-dependent manner (Figure 1). PSO2 demonstrated mostly effective DPPH^●^ inhibition, and it was found that PSO2 at a concentration of 5% *w*/*v* showed significant inhibition of DPPH^●^ radicals when compared with PSO1, COM1 and COM2 (*p* < 0.05). Moreover, linoleic acid (LA) which was the main fatty acid composition in pumpkin seed oil samples also reduced DPPH^●^ radicals in a dose-dependent manner, indicating that LA might exert biological effects in pumpkin seed oil. Although the mechanisms of linoleic acid to react with radicals is still unclear. A previous study by Yu (2001) reported that linoleic acids directly reacted with free DPPH^●^ radicals but had a lag phase and showed no radical quenching activity. However, conjugated linoleic acids reacted and quenched DPPH^●^ radicals in in both hydrophilic and lipophilic environment [51].

**Figure 2:**
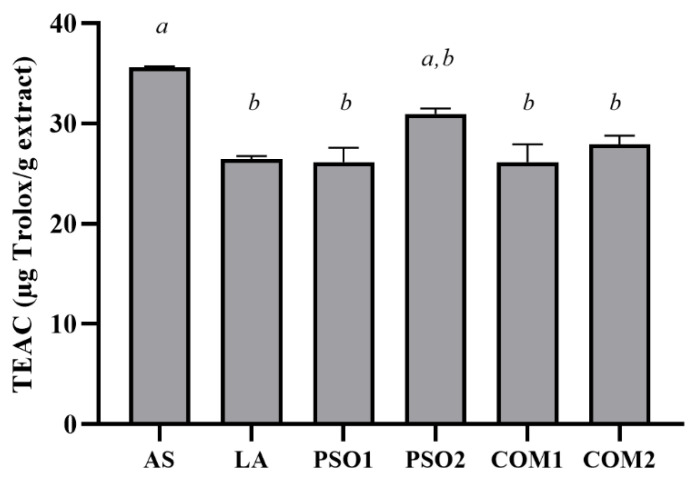
ABTS^●+^ inhibitory effect of pumpkin seed oil samples (PSO1, PSO2, COM1, COM2), linoleic acid (LA), and ascorbic acid (AS). Data obtained from triplicate and three independent experiments are expressed as mean ± SD. The letters, *a* and *b*, presented significant differences among samples in each experiment, *p* < 0.05.

On the other hand, PSO1, PSO2, COM1, and COM2 at a concentration of 10% *w*/*v* decreased ABTS^●+^ radicals. PSO2 showed the highest ABTS^●+^ inhibition when compared with PSO1, COM1, COM2, and LA (*p* < 0.005). Interestingly, PSO2 exhibited comparable ABTS^●+^ inhibition with ascorbic acid, which was a positive control (Figure 2).

**Figure 3:**
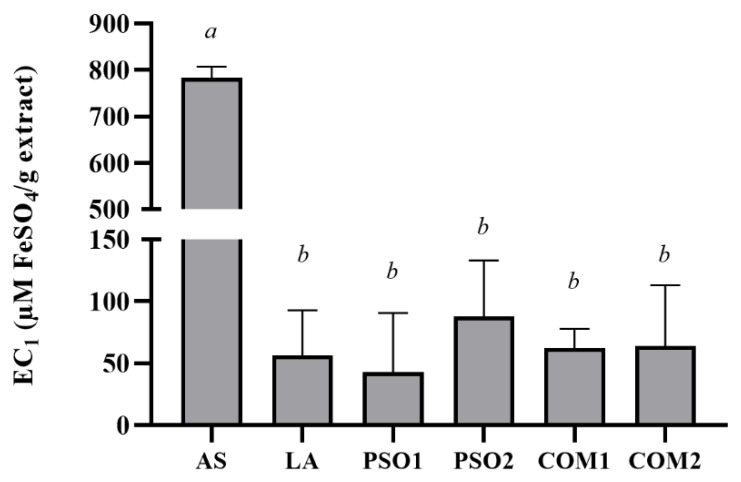
Ferric reducing antioxidant power of pumpkin seed oil samples (PSO1, PSO2, COM1, COM2), linoleic acid (LA), and ascorbic acid (AS). Data obtained from triplicate and three independent experiments are expressed as mean ± SD. The letters, *a* and *b*, presented significant differences among samples in each experiment, *p* < 0.05.

Antioxidant activity of pumpkin seed oil samples based on the antioxidant potential in reducing ferric iron (Fe^3+^) to ferrous iron (Fe^2+^) are shown in Figure 3. This investigation presented the equivalent ferric reducing antioxidant power of PSO1, PSO2, COM1, COM2, and LA at a concentration of 10% *w*/*v*. On the other hand, ascorbic acid, which was a positive control, showed the highest EC_1_ value (*p* < 0.05).

**Figure 4:**
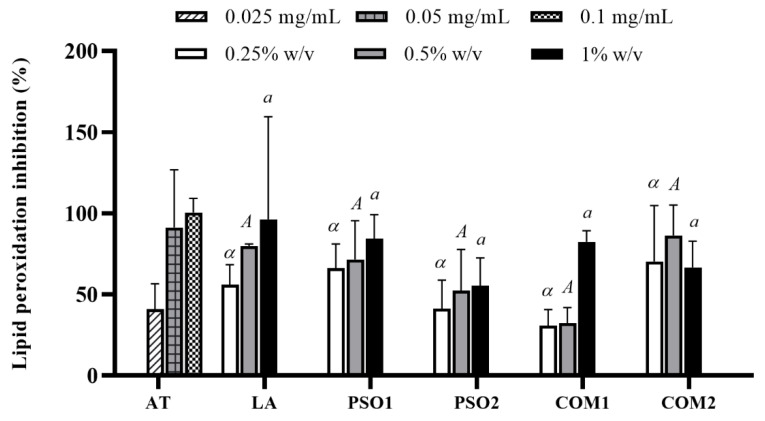
Lipid peroxidation inhibitory effect of pumpkin seed oil samples (PSO1, PSO2, COM1, COM2), linoleic acid (LA), and α-tocopherol (AT). Data obtained from triplicate and three independent experiments are expressed as mean ± SD. The same symbol (*α*) or the same letters (*A* or *a*) denotes no significant difference among samples at the same concentration, *p* < 0.05.

PSO1, PSO2, COM1, COM2 and LA inhibited lipid peroxidation at various concentrations (0.25%, 0.5% and 1% *w*/*v*) in a dose-dependent manner, as shown in Figure 4. The inhibition by LA, PSO1, and COM1 were almost complete at the concentration of 1% *w*/*v*. There was no significantly different inhibition of lipid peroxidation between groups. In addition, α-tocopherol demonstrated effective inhibition on lipid peroxidation at lower concentrations than pumpkin seed oil samples.

Antioxidant activity plays an important role in natural compounds to work against oxidative stress, provide health benefits, and improve some diseases. A considerable number of studies have demonstrated seed oil from a variety of plants contains antioxidants in the form of phenolics, tocopherols, and phytosterols [52,53]. The appearance of polyphenols and carotenoids in pumpkin seed oil stimulated the antioxidant defense system and prevented hypertension, atherosclerosis, type 2 diabetes and cancer [54]. In a previous analysis, pumpkin seed oil was found to have antioxidant activity with Trolox equivalent capacity of 0.664 ± 0.09 to 1.18 ± 0.04 μM Trolox/g [55]. Similarly, Boujemaa I et al. (2020) reported that *C. maxima* showed higher antioxidant activity than *C. moschata* and *C. pepo* [56], which can be described in part by the higher amounts of PUFA, tocopherols and phenolic compounds [18,57]. Recently, antioxidant activities of pumpkin seed oil were measured by four methods including DPPH^●^, ABTS^●^^+^, FRAP and FTC assays. The result demonstrated that PSO2 presented higher value of scavenging activity than PSO1, COM1 and COM2 that significantly reduced the radicals of DPPH^●^ and ABTS^●^^+^, and FeSO_4_. Although PSO2 presented a lower yield of linoleic acid than the others, it exhibited comparable antioxidant activities but with outstanding DPPH^●^ radical scavenging properties. The exerting of scavenging activities of PSO2 might derive from bioactive compounds such as carotenoids, tocopherols and phenolic compounds, supported by prior studies [52,53,54]. It can be concluded that aqueous enzymatic extraction of pumpkin seed oil was a potent extraction method and supported the release of more bioactive components to enhance antioxidant activities. Therefore, all seed oil samples, especially PSO2, demonstrated antioxidant power and might be useful in applications regarding health benefits and medical treatment.

### 3.4. Anti-Aging Activity of Pumpkin Seed Oil

**Figure 5:**
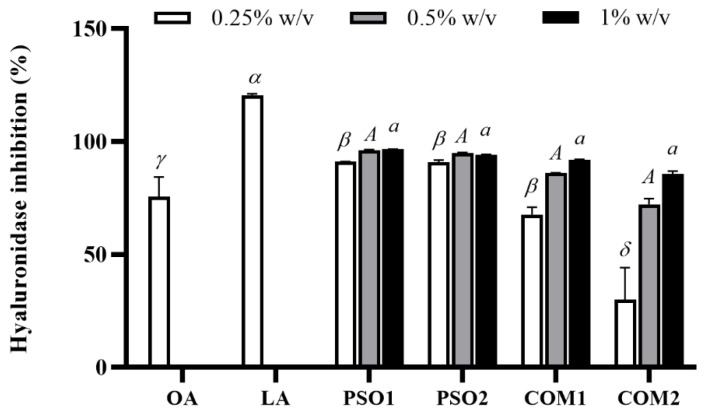
Hyaluronidase inhibitory effect of pumpkin seed oil samples (PSO1, PSO2, COM1, COM2), linoleic acid (LA), and oleanolic acid (OA). Data obtained from triplicate and three independent experiments are expressed as mean ± SD. The symbols, *α*, *β*, *γ*, and *δ*, present significant differences among samples at the concentration of 0.25% *w*/*v*. The capital letter, *A*, presents no significant difference among samples at the concentration of 0.5% *w*/*v*. The letter, *a*, presents no significant differences among samples in at the concentration of 1% *w*/*v*, *p* < 0.05.

PSO1, PSO2, COM1, and COM2 inhibited hyaluronidase activity at various concentrations (0.25%, 0.5% and 1% *w*/*v*) in a dose-dependent manner. At low concentration (0.25% *w*/*v*), PSO1, PSO2, and COM1 showed highly effective inhibition (approximately 70–100%) and significantly inhibited hyaluronidase activity when compared with COM2 (*p* < 0.05) as shown in Figure 5. Oleanolic acid (OA), which was a positive control, also decreased hyaluronidase activity. LA, a main composition of pumpkin seed oil, performed the highest inhibition (120.6 ± 0.7%), which significantly inhibited hyaluronidase activity when compared with other pumpkin seed oil samples and OA (*p* < 0.05).

**Figure 6:**
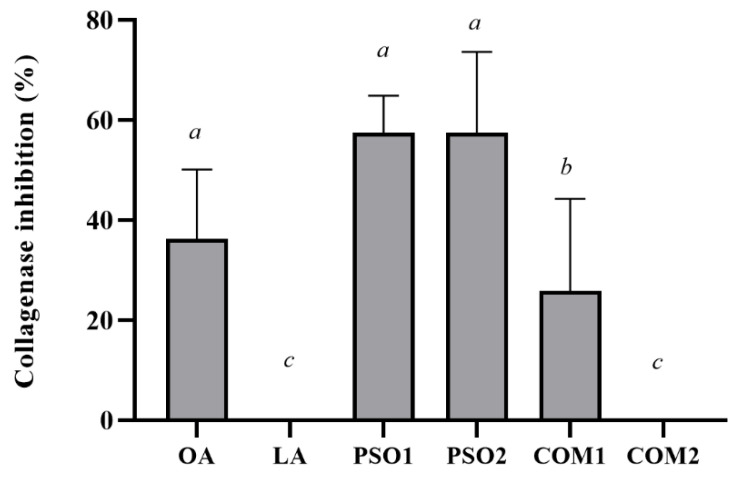
Collagenase inhibitory effect of pumpkin seed oil samples (PSO1, PSO2, COM1, COM2), linoleic acid (LA), and oleanolic acid (OA). Data obtained from triplicate and three independent experiments are expressed as mean ± SD. The letters *a*, *b* and *c* presented significant differences among samples in each experiment, *p* < 0.05.

Anti-collagenase activities of pumpkin seed oil samples are shown in Figure 6. PSO1, PSO2, and COM1 inhibited collagenase activity, while COM2 did not. PSO1 and PSO2 (1% *w*/*v*) displayed significant collagenase inhibitory activity when compared with COM1 and COM2, respectively (*p* < 0.05). OA as positive control also presented inhibition of collagenase activity. Nevertheless, LA did not affect the collagenase in this experiment. This might be the reason why COM2, which contained the highest content of LA, had no effect on collagenase.

**Figure 7:**
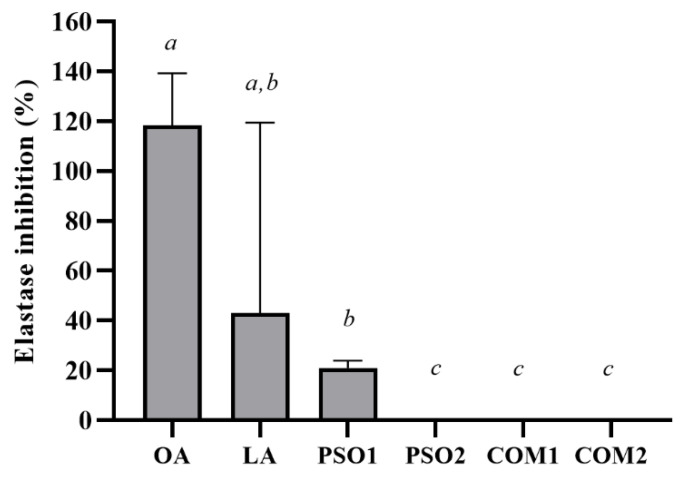
Elastase inhibitory effect of pumpkin seed oil samples (PSO1, PSO2, COM1, COM2), linoleic acid (LA), and oleanolic acid (OA). Data obtained from triplicate and three independent experiments are expressed as mean ± SD. The letters *a*, *b* and *c* presented significant differences among samples in each experiment, *p* < 0.05.

The anti-elastase activities of pumpkin seed oil samples are shown in Figure 7. Only PSO1 at a concentration of 1% *w*/*v* inhibited elastase activity and significantly decreased elastase activity when compared with PSO2, COM1 and COM2 (*p* < 0.05). LA displayed a similar effect with PSO1 and OA. Moreover, OA as positive control presented the highest inhibition of elastase activity and significantly decreased elastase activity when compared with PSO1, PSO2, COM1, COM2, and LA (*p* < 0.05).

Health and beauty of the skin is regarded as one of the most important factors of “well-being” in humans, therefore several anti-aging strategies have been established in recent years [58]. UV irradiation or photoaging induce oxidative stress, which is an important cause for the human skin aging process as well as skin pigmentation [59]. Normally, the human body can generate antioxidant enzymes such as superoxide dismutases (SOD), catalases, and glutathione peroxidase (GSH) to quench reactive oxygen species (ROS) [50], but more antioxidant agents are needed. At present, plant and seed oil extracts have been used for pharmacological and cosmetic products due to their antioxidant properties, which are based on bioactive compounds including phenolics, tocopherols and phytosterols, resulting in improved skin aging [23,60]. Previous research found that *Camellia japonica* seed oil induced human type I collagen synthesis with a high moisturizing effect; nevertheless, it inhibited MMP1 activity [61]. Pomegranate seed oil enriched PUFA and antioxidant effect and their products; nanoemulsions and creams improved skin barrier function [62]. However, there has been little research performed on the cosmetic properties of pumpkin seed oil, consequently, this study focused on determining the inhibition of hyaluronidase, collagenase, and elastase enzymes involving anti-aging activities. The results found that PSO1 and PSO2 potentially inhibited hyaluronidase enzyme as well as COM1 and COM2, which showed similar effects to inhibit hyaluronidase activity. Additionally, PSO1 and PSO2 significantly decreased collagenase enzyme activity, with COM1 slightly reducing the enzyme activity. Only PSO1 performed the inhibition of the elastase enzyme. Thus, it can be determined that the aqueous enzymatic extraction of pumpkin seed oil (PSO1 and PSO2) had more potent anti-aging activities showing anti-hyaluronidase, anti-collagenase, and anti-elastase activities than cold-press of pumpkin seed oil (COM1 and COM2), which correlate with their antioxidant activities. To sum up, the results supported that PSO1 and PSO2 show effective anti-aging effects and could be applied to skin cosmetics.

### 3.5. Anti-Tyrosinase Activity of Pumpkin Seed Oil

**Figure 8:**
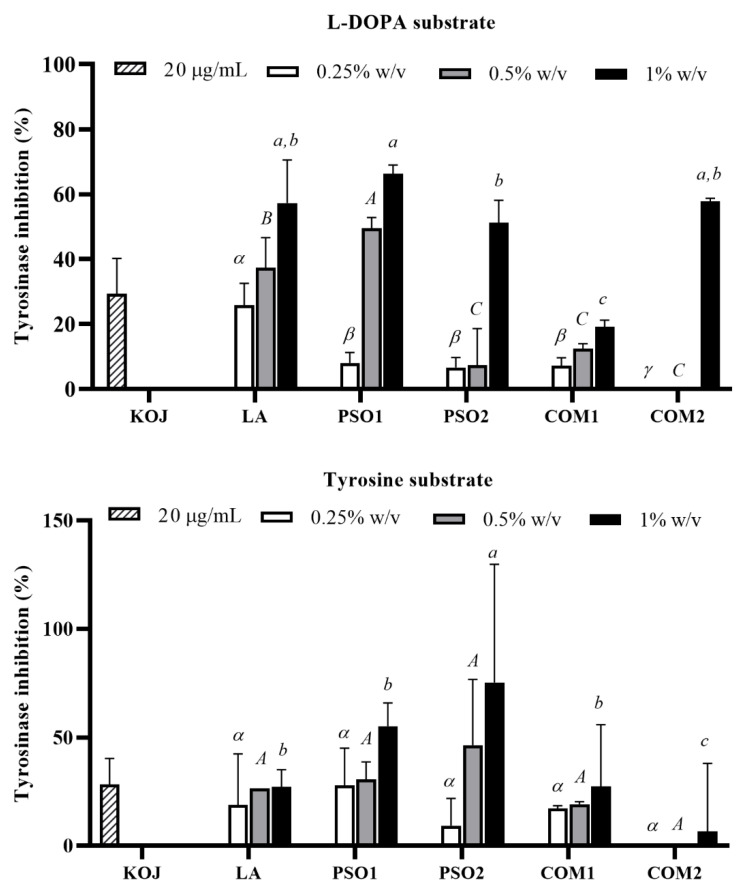
Tyrosinase inhibitory effect of pumpkin seed oil samples (PSO1, PSO2, COM1, COM2), linoleic acid (LA), and kojic acid (KOJ) when the substrate was L-DOPA and tyrosine. Data obtained from triplicate and three independent experiments are expressed as mean ± SD. The symbols, *α*, *β*, and *γ*, present significant differences among samples at the concentration of 0.25% *w*/*v*. The capital letters, *A*, *B*, and *C*, present significant differences among samples at the concentration of 0.5% *w*/*v*. The letters, *a*, *b*, and *c*, present significant differences among samples at the concentration of 1% *w*/*v*, *p* < 0.05.

Hyperpigmentation is another skin problem which can occur after UV exposure. Melanogenesis plays an important role in skin pigmentation which is controlled by the action of the tyrosinase enzyme to product the melanin pigment. Initially, L-tyrosine is hydroxylated by tyrosinase and converted to L-3,4-dihydroxyphenylalanine (L-DOPA), which is then oxidized to DOPA-quinone and finally to melanin pigments [63]. Anti-tyrosinase activities when L-DOPA and tyrosine were used as substrates of pumpkin seed oil samples are presented in Figure 8. PSO1, PSO2, COM1, and COM2 inhibited tyrosinase activity which used L-DOPA as a substrate in a dose-dependent manner, except COM2. Obviously, PSO1 showed the highest tyrosinase inhibitory activity at a concentration of 0.5% and 1% *w*/*v* when compared with PSO2, COM1, and COM2 (*p* < 0.05). Likewise, all pumpkin seed oil samples inhibited tyrosinase activity when tyrosine was used as a substrate. The inhibitions were in a dose-dependent manner, except COM2. PSO2 showed the highest tyrosinase inhibitory activity at a concentration of 0.5% and 1% *w*/*v* when compared with PSO1, COM1, and COM2 (*p* < 0.05). Similarly, LA performed tyrosinase inhibition in a dose-dependent manner, and kojic acid (positive control) also decreased tyrosinase activity in both substrates. It was indicated that PSO1 and PSO2 had potent anti-tyrosinase activities, supporting their use in applications for whitening skin.

Currently, several researchers have investigated many natural compounds used as ingredients for skin whitening products [64]. Tea seed oil, which is rich in oleic acid and antioxidant power, inhibited tyrosinase and TPR-2 activities, leading to the suppression of the process of melanogenesis [65]. Moreover, the report from Hong Xin Cui, et al. (2018) found that seed oil from *Torreya grandis* revealed potent antioxidant activity to inhibit tyrosinase activity, via the reduction of the oxygen supply in the tyrosinase reaction [66]. Our findings demonstrated that pumpkin seed oil exhibited tyrosinase enzyme activity. PSO1 presented significant inhibition of tyrosinase by using L-DOPA as a substrate. PSO2 reported significant inhibition of tyrosinase by using tyrosine as a substrate. Our results indicated aqueous enzymatic extraction of pumpkin seed oil (PSO1 and PSO2) also has powerful anti-tyrosinase activities for whitening effects through their antioxidant activity.

## 4. Conclusions

Oils from *C. moschata* seeds were successfully extracted by aqueous enzymatic extraction. Linoleic acid was the main composition in all *C. moschata* seed oil samples, but lower amounts were detected in PSO1 and PSO2 compared with the commercial oils. *C. moschata* seed oils extracted by aqueous enzymatic extraction (both PSO1 and PSO2) possessed comparable antioxidant activities but superior inhibitory effects on collagenase, hyaluronidase, and tyrosinase as compared to commercial pumpkin seed oils. Therefore, aqueous enzymatic extraction of pumpkin seed oil proves to be useful for containing simultaneous oil and bioactive compounds, showing to be a more environmentally friendly alternative to solvent extraction. Furthermore, PSO1 and PSO2 were suggested as good sources of nutrients and bioactive compounds, which can be alternatively used in functional food and cosmetic industries.

## Figures and Tables

**Table 1:** Fatty acid compositions in pumpkin seed oil samples.

Fatty Acid Composition (%)	Pumpkin Seed Oil			
	PSO1	PSO2	COM1	COM2
**Unsaturated fatty acid**				
Palmitoleic acid (C16:1)	0.09	0.14	0.08	0.09
Oleic acid (C18:1 omega-9)	31.22	37.45	33.39	28.64
Cis linoleic acid (C18:2 omega-6)	39.09	37.63	48.00	51.74
Alpha linolenic acid (C18:3 omega-3)	0.14	0.03	0.28	0.22
Eicosenoic acid (C20:1 omega-9)	0.09	0.08	0.06	0.05
Docosahexaenoic acid (C22:6 omega-3)	0.01	ND	ND	ND
**Total monounsaturated fatty acid**	**31.40**	**37.67**	**33.53**	**28.78**
**Total polyunsaturated fatty acid**	**39.24**	**37.66**	**48.28**	**51.96**
**Saturated fatty acid**				
Lauric acid (C12:0)	0.01	0.02	ND	ND
Myristic acid (C14:0)	0.10	0.19	0.08	0.09
Palmitic acid (C16:0)	19.08	13.47	12.17	12.36
Heptadecanoic acid (C17:0)	ND	ND	0.05	0.08
Stearic acid (C18:0)	9.37	10.98	5.25	6.05
Arachidic acid (C20:0)	0.37	0.91	0.31	0.35
Behenic acid (C22:0)	0.07	0.20	0.08	0.09
Lignoceric acid (C24:0)	0.03	0.12	0.10	0.09
**Total saturated fatty acid**	**29.03**	**25.89**	**18.04**	**19.11**

ND = Not detected; PSO1 = pumpkin seed oil from *Cucurbita moschata* Duch. Ex Poir.; PSO2 = pumpkin seed oil from *Cucurbita moschata* (Japanese pumpkin); COM1 = commercial pumpkin seed oil sample 1; COM2 = commercial pumpkin seed oil sample 2.

## Data Availability

All data, tables and figures in this manuscript are original.
